# Male clients of male sex workers in West Africa: A neglected high-risk population

**DOI:** 10.1371/journal.pone.0212245

**Published:** 2019-05-01

**Authors:** Cheick Haïballa Kounta, Luis Sagaon-Teyssier, Pierre-Julien Coulaud, Marion Mora, Gwenaelle Maradan, Michel Bourrelly, Abdoul Aziz Keita, Stéphane-Alain Babo Yoro, Camille Anoma, Christian Coulibaly, Elias Ter Tiero Dah, Selom Agbomadji, Ephrem Mensah, Adeline Bernier, Clotilde Couderc, Bintou Dembélé Keita, Christian Laurent, Bruno Spire, the CohMSM Study Group

**Affiliations:** 1 Aix Marseille Univ, INSERM, IRD, SESSTIM, Sciences Economiques & Sociales de la Santé & Traitement de l’Information Médicale, Marseille, France; 2 ORS PACA, Observatoire régional de la santé Provence-Alpes-Côte d’Azur, Marseille, France; 3 ARCAD Sida, Bamako, Mali; 4 Espace Confiance, Abidjan, Côte d’Ivoire; 5 Association Africaine Solidarité, Ouagadougou, Burkina Faso; 6 Centre Muraz, Bobo-Dioulasso, Burkina Faso; 7 Espoir Vie Togo, Lomé, Togo; 8 Coalition Internationale Sida, Pantin, France; 9 IRD, INSERM, Univ Montpellier, TransVIHMI, Montpellier, France; David Geffen School of Medicine at UCLA, UNITED STATES

## Abstract

Research on male clients of male sex workers (MCMSW) has been neglected for a long time globally. We aimed to characterize MCMSW and to identify factors associated with their sexual practices using data from the prospective cohort study CohMSM conducted in Burkina Faso, Côte d’Ivoire, Mali and Togo. Our study focused on HIV-negative men who have sex with other men (MSM), recruited between 06/2015 and 01/2018 by a team of trained peer educators. Scheduled study visits at 6, 12 and 18 months included medical examinations, HIV screening, risk-reduction counselling and face-to-face interviews to collect information on their sociodemographic characteristics, sexual behaviours, and HIV risk-reduction strategies (HIV-RRS). Three stigmatization sub-scores were constructed (experienced, perceived and internalized). Mixed-effects logistic regression was used for data analysis. Of the 280 participants recruited at baseline, 238, 211 and 118, respectively, had a follow-up visit at 6, 12 and 18 months. Over a total of 847 visits, 47 transactional sex (TS) encounters were reported by 38 MCMSW (13.6%). Of the latter, only one participant reported systematic TS (2.6%), 18 (47.4%) stopped reporting TS after baseline, while 6 (15.8%) reported TS after baseline. Thirteen participants (34.2%) reported occasional TS. After adjusting for country of study and age, the following self-reported factors were associated with a greater likelihood of being MCMSW: protected anal sex, exclusively insertive anal sex with male sexual partners, avoidance of sex after consuming psychoactive products and experiencing stigmatization (all during the previous 6 months). The majority of MCMSW in this study practiced HIV-RRS with male sexual partners, including engaging in protected anal sex, avoidance of sex when consuming psychoactive products, and practising exclusively insertive anal sex.

## Introduction

Men who have sex with other men (MSM), including male sex workers (MSW), are at a much greater risk of acquiring and transmitting sexually transmitted infections (STI), including human immunodeficiency virus (HIV). This is particularly true in Sub-Saharan Africa, where the prevalence of HIV infection is estimated at 36.3% in MSW compared with 17% in the general MSM population [1–7]. Despite the vast literature on sex work, most studies to date have focused exclusively on female sex workers [8–12]. Of the studies focusing on MSW, few have investigated the risk of HIV/STI transmission [13,14,2,15,16], and research on male clients of MSW (MCMSW) is very scarce [17–20].

In Africa, MCMSW constitute a highly heterogeneous group comprising bisexuals, transgender people, homosexuals and men who consider themselves heterosexual but who have sex with men for a variety of reasons (e.g. isolation, economic compensation, sexual desire, and typical ideals of gender) in a context where consensual sexual relations between adults of the same sex are strongly stigmatized and socially marginalized [21–23]. In addition, these relationships are penalized by law in some African countries, exposing them to the risk of arbitrary arrest, prosecution and imprisonment [24,25]. These discriminatory laws contribute to the perpetuation of stigma and discrimination, as well as hate crimes, violence, torture and ill-treatment, police abuse, domestic and community violence, and constitute a barrier to health equity by preventing access to health services including HIV-related services, which in turn may influence individuals’ level of risk of HIV exposure and transmission [26–28].

Despite being a very neglected high risk group, MCMSW are of key importance in the fight against HIV, given their potential role as a bridge for HIV transmission to the general population [17,18,29,30]. A substantial proportion of MCMSW in Africa marry women to avoid a hostile anti-MSM social environment [31,32]. In this way, they can continue to have sexual relations (commercial or not) outside of marriage with male partners. Their non-commercial partners (wife and/or steady male partners) are often unaware of their spouse’s/partner’s commercial sex activities, and the fact that they are being put at greater risk of exposure to HIV. A few studies have highlighted that a substantial proportion of MSM who pay for sex and/or have been paid for sex [19,33,34] have risky sexual behaviours, especially condomless insertive or receptive anal sex [35,18,17].

In this study, our main hypothesis was that MCMSW have risk factors for HIV infection which are associated with their sexual practices. Characterizing MCMSW is crucial in order to investigate not only whether a particular profile exists or not for this sub-population, but also to have a better understanding about their psychosocial characteristics and their sexual behaviours. This study had two objectives: first, to compare MCMSW with the general MSM population in terms of their sociodemographic and economic characteristics (e.g., age, educational level, employment status, income) and psychosocial characteristics (e.g. self-defined sexual and gender identities, sudden sexual violence by male partners); second, to identify factors associating MCMSW with TS. The study’s findings will be useful for healthcare providers and researchers because they offer the first comprehensive insight into both MCMSW and the HIV and STI exposure factors associated with this sub-population’s sexual practices.

## Materials and methods

### CohMSM study procedures

In June 2015, a prospective cohort study of MSM was initiated at the premises of four local community-based organisations providing HIV services to MSM in four West African cities: Abidjan (Côte d’Ivoire), Bamako (Mali), Lomé (Togo) and Ouagadougou (Burkina Faso). Its main objectives were to assess both the feasibility and value of providing novel three-monthly preventive global care for MSM in West Africa, in order to help reduce the incidence of HIV in this key population, in their female partners and in the general population. The study did not compare a control group with an exposed group, nor was it based on a clinical trial. Potential participants were identified and recruited by a team of trained peer educators from these local organisations who approached individuals through a specific MSM network. Eligibility criteria included being at least 18 years old, and reporting at least one anal sexual intercourse (insertive or receptive) with another man in the 3 months preceding study enrolment. Eligible individuals were offered a quarterly preventive global care package including: i) collection of information on health status, STI symptoms and sexual behaviours of individuals, ii) clinical examination, iii) diagnosis of STI and if necessary their treatment, iv) prevention tips tailored to MSM based on risk-reduction counselling, and v) provision of condoms and lubricants. In addition, vaccination against hepatitis B and annual tests for syphilis were proposed. HIV-negative MSM were also offered an HIV test at each quarterly visit. MSM found to be HIV-positive were offered immediate care for their infection, including ARV treatment. Before starting the interview, participants systematically received detailed information about the survey’s objectives and their right to interrupt the interview without justification. At enrolment and follow-up visits, participants completed face-to-face interviews with a research assistant who collected information on their sociodemographic and economic characteristics, HIV risk-reduction strategies, alcohol consumption, drug use and stigmatization. Participants had to provide written informed consent. The study team was very attentive to ensuring anonymity and confidentiality. Ethics approval was obtained from the National Ethics Committees of Mali (N°2015/32/CE/FMPOS), Burkina Faso (N°2015-3-037), Côte d’Ivoire (N°021/MSLS/CNER-dkn) and Togo (N°008/2016/MSPSCAB/SG/DPML/ CBRS). The study protocol was designed in accordance with the ethical charter for research in developing countries of French National Agency for Research on AIDS and Viral Hepatitis (ANRS) in France. The ClinicalTrials.gov Identifier is NCT02626286.

### Study population

Between 06/2015 and 01/2018, 778 participants were enrolled in CohMSM. All HIV-positive participants (n = 154), participants receiving benefits for sex (i.e. money, accommodation or gifts) at least once during the follow-up (n = 294) and those who did not complete a sociodemographic questionnaire at baseline (n = 50) were excluded from the present analysis (S1 Appendix). The present analysis therefore focused on the remaining 280 HIV-negative MSM of whom 238, 211 and 118, respectively, had a follow-up visit at 6- and 12- and 18-months.

### Variables

#### Outcome

The outcome of this study was constructed on the basis of the following question: "During the last 6 months, have you been in a situation where you gave money, accommodation or any other benefit in exchange for sex with a man?”. Participants who responded “always” or “sometimes”, in contrast to those who responded “never”, were categorized as MCMSW. This question was asked at all follow-up visits. MCMSW who had also received benefits for sex with MSM at least once during the follow-up were excluded from the present analysis.

#### Explanatory variables

*a)* Socio-demographic and economic characteristics: age was specified as a continuous variable. Dichotomous variables were constructed to indicate whether participants had at least a high-school level of education (= 1 vs. < high-school = 0), were married or cohabitating (= 1 vs. single, divorced or widowed = 0), and whether they had a stable housing status (= 1 vs. unstable housing status = 0). Socio-economic characteristics included employment status (employed = 1 vs. unemployed = 0), monthly income dichotomised at the median (50 000 Francs de la Communauté Financière en Afrique, approximately US$89.28 in 2017), source of income (work = 1 or aid = 0) and self-perceived financial situation (comfortable = 1 vs. difficult = 0).

b) Sexual characteristics: a dichotomous variable indicated self-defined gender identity (both a man and a woman = 0 vs. man exclusively = 1), and a self-defined sexual orientation identity variable, indicated whether participants perceived themselves to be bisexual (= 0 vs. not bisexual including homosexual, heterosexual = 1). These gender and sexual identity variables are similar to those used in other studies [26,36,31]. A dichotomous avoidance variable was constructed (no = 0 vs. yes = 1) to indicate whether participants practiced HIV risk-reduction strategies (e.g., avoiding sexual relations when drunk or when consuming other psychoactive products; using antiretroviral drugs to reduce the risk of HIV infection; avoiding anal penetration by seropositive partners or partners of unknown serostatus, etc.) (S2 Appendix). Sexual behaviour was recorded using various variables: i) sexual position taken with male partners (exclusively insertive = 0 vs. receptive or versatile = 1 and not documented = 2); ii) condom use with male partners during anal sex (no = 0 vs. yes = 1), iii) condom use with male partners during oral sex (no = 0 vs. yes = 1), iv) number of male sexual partners (more than one = 1 vs. one = 0), v) disagreement about condom use with male partners (no = 0 vs. yes = 1), vi) group sex with men (no = 0 vs. yes = 1). The information provided by all these variables concerned the 6 months before the survey. Another variable, entitled “searching for male sexual partners on the internet” (no = 0 vs. yes = 1), concerned the previous 4 weeks.

c) Stigmatization during the previous 6 months: three sub-scores were constructed ranging from 0 to 10. They were based on items taken from previous study (S3 Appendix) [37]: 1) “experienced stigmatization” (based on 5 items, Cronbach’s alpha = 0.58); 2) “perceived stigmatization” (based on 11 items, Cronbach’s alpha = 0.70); and 3) “internalized stigmatization” (based on 8 items, Cronbach’s alpha = 0.80).

Socio-demographic and economic characteristics were measured at baseline only and were specified in the model as time-fixed variables. In contrast, sexual behaviour and stigmatization variables were measured at each time-point in the follow-up and consequently were specified as time-varying.

### Statistical analysis

Descriptive analysis was conducted to compare baseline socio-demographic and economic characteristics, and sexual behaviours of MSM practising TS as clients (i.e. MCMSW) and MSM who did not. Categorical variables were compared between these two groups using Pearson's chi-squared test (χ2). Continuous variables were compared using Student’s t-test.

Univariate and multivariate analyses were then performed using mixed-effects logistic regression to account for the correlation of repeated data over time. All explanatory variables were first tested in univariate mixed-effects logistic regression. Potential variables for the multivariate model were then selected with a p-value<0.2. The final multivariate model was estimated using a forward procedure, which consisted in placing all candidate variables into the multivariate model for testing one by one, and then retaining those with a p-value < 0.05. Fixed effects for each study country were specified in order to avoid bias arising from differences in sample sizes. All statistical analyses were performed using Stata version 13.0 (Stata Corp, College Station, Texas, USA).

## Results

### Overall sample description

Of the 280 HIV-negative participants in this analysis, 238, 211 and 118 had a follow-up visit at 6- and 12- and 18-months, respectively. Over a total of 847 visits, 47 TS encounters were reported by 38 MCMSW (13.6%). Of the latter, only one participant reported systematic TS (2.6%), 18 (47.4%) stopped reporting TS after baseline, and 6 (15.8%) reported TS after baseline. Thirteen participants (34.2%) reported occasional TS.

**Table 1** shows the comparative analysis of baseline individual characteristics between MCMSW and non-MCMSW. The former were significantly older (average age of 28.5 years vs. 25.4 years, p<0.0017). Furthermore, 68.4% (vs. 51.2%) of them were significantly more likely to have an educational level < high-school diploma. A majority of MCMSW (84.2% vs. 76.9%) were unmarried (single, divorced or widowed) and although 47.4% had an income generating activity, 71.1% reported their financial situation as difficult. Fifty-four percent (52.6%) had a monthly income above the median (50 000 FCFA). Furthermore, MCMSW were significantly more likely (73.7% increased probability) to report work (as opposed to financial aid) as the main source of their income (p = 0.015). Despite having work, they were significantly more likely (50% increased probability, p = 0.004) to have unstable housing. Almost half of the MCMSW (47.4%) defined themselves as bisexual while 71.1% identified themselves as being men or boys. MCMSW were also significantly more likely to have exclusively insertive anal sex with male partners.

**Table 1:** Comparative analysis of the baseline characteristics of the study sample (n = 280).

Sociodemographic and economic characteristics	Male Clients n = 38 (13.6%) n (%)	No Male Clients n = 242 (86.4%) n (%)	p Value^*a*^
Study country (n = 280)			
Mali	15 (39.5)	62 (25.6)	**0.006**
Cote d'Ivoire	4 (10.5)	65 (26.9)	
Burkina	5 (13.2)	66 (27.3)	
Togo	14 (36.8)	49 (20.3)	
Age (n = 280)			
Age [mean ± standard deviation]	[28.5±7.7]	[25.4±5.2]	**0.0017**
Education level (n = 280)			
≥ high-school diploma	12 (31.6)	118 (48.8)	**0.048**
< high-school diploma	26 (68.4)	124 (51.2)	
Marital status (n = 280)			
Married or living in a couple	6 (15.8)	56 (23.1)	0.310
Single, Divorced, Widowed	32 (84.2)	186 (76.9)	
Had an income generating activity (n = 280)			
No	20 (52.6)	153 (63.2)	0.212
Yes	18 (47.4)	89 (36.8)	
Monthly income relative to the median (n = 275)			
≤median (50 000 Fcfa)	18 (47.4)	141 (59.5)	0.160
>median (50 000 Fcfa)	20 (52.6)	96 (40.5)	
Sources of income (n = 280)			
Aid	10 (26.3)	115 (47.5)	**0.015**
Work	28 (73.7)	127 (52.5)	
Financial perception (n = 280)			
Comfortable	11 (28.9)	87 (35.9)	0.400
Difficult	27 (71.1)	155 (64.1)	
Stable housing (n = 280)			
Yes	19 (50.0)	177 (73.1)	**0.004**
No	19 (50.0)	65 (26.9)	
Self-defined sexual identity (n = 280)			
Bisexual	18 (47.4)	129 (53.3)	0.496
Not bisexual	20 (52.6)	113 (46.7)	
Self-defined gender identity (n = 276)			
Both a man and woman	11 (28.9)	89 (37.4)	0.314
Man or Boy	27 (71.1)	149 (62.6)	
Sexual positioning with male partners in the previous 6 months			
Receptive or versatile	15 (39.5)	140 (57.9)	**0.043**
Exclusively insertive	23 (60.5)	96 (39.7)	
ND^b^	0 (0.0)	6 (2.5)	

^a^p Calculated with Pearson's chi-squared test (χ2) for categorical variables, Student’s t-test for continuous variables.

^b^ND = not documented. Includes missing data, “does not know” and “no response” terms.

### Factors associating MCMSW with transactional sex

Results from the multivariate analysis (**Table 2**)—after adjusting for the four study countries—showed that the probability of being an MCMSW increased by 4.8% per 1-year increase in age [adjusted odds ratio (aOR) and 95% confidence interval (95% CI):1.048 (1.00–1.10)]. Furthermore, the more participants experienced stigmatization, the higher the probability was that they were MCMSW (this increase reaching 92.0%) (aOR [95%CI]:1.920[1.31–2.81]) during the previous 6-months. In terms of HIV-RRS, participants who self-reported that they practised protected anal sex with male partners were twice as likely to be MCMSW (aOR [95%CI]:2.211[1.15–4.24]) than those who did not report HIV-RRS. Participants who self-reported avoiding sex when drunk or when consuming psychoactive products were 8 times more likely to be MCMSW (aOR [95%CI]:8.789[1.15–67.20]). Finally, participants who self-reported that they exclusively practised insertive anal sex with male sexual partners in the previous 6 months were more than twice as likely to be MCMSW (aOR [95%CI]:2.257[1.12–4.53]).

**Table 2:** Factors associated with male clients of male sex workers in West Africa: univariate and multivariate analyses with mixed effect logistic regression(n = 280, 847 follow-up visits).

Background characteristics	Follow up visits		Univariate analysis^*a*^		Multivariate analysis^*b*^	
	Client	No Client				
	n = 47 (100%)	n = 800 (100%)	OR [95% CI]^*c*^	p^*e*^	aOR [95% CI]^d^	p^*e*^
Follow-up time (N = 847)						
At baseline	24 (51.1)	256 (32.0)				
At 6 months	8 (17.0)	230 (28.8)				
At 12 months	7 (14.9)	204 (25.5)				
At 18 months	8 (17.0)	110 (13.8)				
Study country						
Mali	19 (40.4)	240 (30.0)	Ref		Ref	
Cote d'Ivoire	4 (8.5)	196 (24.5)	0.251 [0.08–0.75]	**0.014**	0.209 [0.07–0.66]	**0.007**
Burkina	9 (19.2)	203 (25.4)	0.568 [0.25–1.29]	0.177	0.571 [0.23–1.40]	0.219
Togo	15 (31.9)	161 (20.1)	1.252 [0.60–2.60]	0.547	0.960 [0.45–2.06]	0.916
Age						
Age	47 (100)	800 (100)	1.049 [1.01–1.09]	**0.021**	1.048 [1.00–1.10]	**0.054**
Education level						
≥ high-school diploma	17 (36.2)	371 (46.4)	Ref			
< high-school diploma	30 (63.8)	429 (53.6)	1.546 [0.84–2.86]	0.164		
Monthly income relative to the median						
≤median (50 000 Fcfa)	22 (46.8)	444 (56.9)	Ref			
>median (50 000 Fcfa)	25 (53.2)	337 (43.1)	1.495 [0.83–2.71]	0.184		
Sources of income						
Aid	13 (27.7)	355 (44.4)	Ref			
Work	34 (72.3)	445 (55.6)	2.092 [1.08–4.03]	**0.028**		
Stable housing						
Yes	28 (59.6)	570 (71.2)	Ref			
No	19 (40.4)	230 (28.8)	1.698 [0.93–3.11]	0.087		
Self-defined gender identity						
Both a man and woman	9 (19.1)	281 (35.3)	Ref			
Man or boy	38 (80.9)	515 (64.7)	2.332 [1.11–4.91]	**0.026**		
Sexual positioning with male partners in the previous 6 months						
Receptive or versatile	16 (34.1)	397 (49.6)	Ref		Ref	
Exclusively insertive	30 (63.8)	324 (40.6)	2.344 [1.25–4.39]	**0.008**	2.257 [1.12–4.53]	**0.022**
ND^f^	1 (2.1)	79 (9.8)	0.341 [0.04–2.65]	0.304	0.665 [0.08–5.52]	0.705
Condom use with male partners during anal sex during the previous 6 months						
No	19 (40.4)	458 (57.2)	Ref		Ref	
Yes	28 (59.6)	342 (42.8)	1.887 [1.03–3.46]	**0.040**	2.211 [1.15–4.24]	**0.017**
Condom use with male partners during oral sex during the previous 6 months						
No	33 (70.2)	455 (56.9)	Ref			
Yes	14 (29.8)	345 (43.1)	0.582 [0.31–1.11]	0.099		
Disagreement about condom use with male partners during the previous 6 months						
No	36 (76.6)	720 (90.0)	Ref			
Yes	11 (23.4)	80 (10.0)	2.518 [1.21–5.23]	**0.013**		
Number of male sexual partners during the previous 6 months						
One	11 (23.4)	331 (41.4)	Ref			
More than one	36 (76.6)	469 (58.6)	2.239 [1.12–4.48]	**0.023**		
Searched for male sexual partners on the internet during the previous 4 weeks						
No	25 (53.2)	528 (66.0)	Ref			
Yes	22 (46.8)	272 (34.0)	1.696 [0.94–3.07]	0.082		
Group sex with men						
No	38 (80.9)	731 (91.4)	Ref			
Yes	9 (19.1)	69 (8.6)	2.146 [0.90–5.12]	0.085		
**HIV risk-reduction strategies practiced**						
avoiding anal penetration of seropositive partners or partners whose serostatus was unknown						
No	5 (10.6)	191 (23.9)	Ref			
Yes	42 (89.4)	609 (76.1)	2.792 [1.08–7.19]	**0.033**		
avoiding anal penetration by seropositive partners or partners whose serostatus was unknown						
No	4 (8.5)	190 (23.8)	Ref			
Yes	43 (91.5)	610 (76.2)	3.551 [1.25–10.06]	**0.017**		
avoiding sexual relations when drunk or when consuming other psychoactive products, in order to reduce the risk of HIV infection						
No	1 (2.1)	131 (16.4)	Ref		Ref	
Yes	46 (97.9)	669 (83.6)	10.024 [1.36–73.74]	**0.024**	8.789 [1.15–67.20]	**0.036**
**Stigmatization Scores**						
Experienced stigmatization during the previous 6 months	47 (100)	800 (100)	1.477 [1.05–2.08]	**0.025**	1.920 [1.31–2.81]	**0.001**

^a^Univariate analysis using a mixed-effect logistic regression model.

^b^Multivariate analysis using a multivariate stepwise mixed effect logistic regression.

^c^OR = odds ratio; CI = confidence interval.

^d^aOR = adjusted odds ratio; CI = confidence interval.

^e^p Calculated with Wald chi2 test.

^f^ND = not documented. Includes missing data, “does not know” and “no response” terms.

## Discussion

Despite the several studies conducted to date on male clients of Female Sex Workers [38–41], and the growing amount of literature exploring the experiences and practices of male sex workers (MSW) [14,32,42,43], few studies have focused on male clients of male sex workers (MCMSW). To our knowledge, this is the first to investigate the issue of transactional sex (TS) in MCMSW in West Africa. Our findings add to the literature by providing information regarding sociodemographic and economic characteristics of this population in four West African countries.

Approximately 14% of our study population were MCMSW (n = 38). This percentage is much higher than that found in a study in China (5%) [17]. The majority of the MCMSW in our study reported employing risk reduction strategies (RRS) with male sexual partners, including engaging in protected anal sex, and avoidance of sex when consuming psychoactive products. Similar results for the use of RRS among MCMSW were documented in a study in Vietnam in 2013, which first evaluated participants’ perception of risk and then the RRS they implemented before sexual intercourse [44]. Future intervention efforts should encourage existing HIV RRS among MCMSW. Although the subject of a ‘causal relationship’ between homosexuality, unsafe sex and HIV infection has dominated HIV and AIDS discourse in the West [45,46], our findings suggest that pressures, roles and power related to TS may influence the ability of men to behave in ways that reflect their risk perceptions. Accordingly, our results for MCMSW provide some support for health behaviour models which posit that greater perceived risk is associated with fewer risky sexual behaviours. HIV/AIDS education and prevention programs should investigate in greater detail how TS in key populations such as MSM may influence risk perceptions and risk behaviours. Successful HIV prevention strategies for hard-to-reach MCMSW populations require effective integration of evidence-based biomedical, behavioural, and structural interventions, especially in the African context where HIV prevention is centred more on heterosexual contact and vaginal intercourse. As well as social norms, erroneous representations of HIV risks and taboos, HIV prevention programs should also take into account all forms of sexuality and social status [44,47,48].

We found that participants who self-reported that they exclusively had insertive anal sex with male sexual partners in the previous 6 months, were more likely to be MCMSW. Previous research showed that insertive anal sex is less risky for HIV contamination than receptive or versatile anal sex [49,50]. In our study, the practice of insertive anal sex reported by MCMSW might well contribute to reduce the spread of HIV within and by this population, given that TS is a known factor for increased likelihood of HIV transmission [13]. Globally, safer TS plays an important role in risk reduction of HIV and other STI not only for men but also for women [51].

This was further indicated by our findings that the probability of being MCMSW tended to increase for each 1-year increase in age. This result contrasts with prior research in the US, China and Australia [19,17,30]. The older age of MCMSW might be a barrier to finding younger regular sex partners, which in turn may push them to engage more in transactional sex [52]. Finally, our findings also indicated that the more participants experienced stigmatization, the higher the probability was that they were MCMSW. West Africa is hostile in general to homosexuality, but our results do not provide information as to why MCMSW seem to experience more stigmatization than other MSM [22,25–27,32].

The primary strengths of our study come from the fact that the CohMSM study was performed in four West African countries, was longitudinal in nature, and had four scheduled visits. Some study limitations should be taken into account when interpreting our results. First, the CohMSM study was designed to examine the feasibility of introducing a preventive intervention among MSM, and not specifically to explore the issue of MCMSW. Second, given the declarative nature of the data and the fact that respondents participated in face-to-face interviews, social desirability bias is possible. Accordingly, sexual risk behaviours may have been underreported. However, this bias was perhaps minimized by the fact that the research assistants involved all worked close to the ground, came from recognized non-governmental organizations, and were directly involved with the MSM population. It is likely therefore that a trustful relationship emerged over time with the research assistants, and consequently, that they were able to accurately identify MCMSW.

Despite the study’s limitations, our results suggest that MCMSW should be provided with long-term HIV prevention interventions which: (1) focus on individual behaviour change (addressing barriers to condom use with other alternative means of prevention such as pre-exposure prophylaxis (PrEP), enhancing current risk reduction practices); (2) incorporate interpersonal contexts (simultaneously engaging MCMSW and their peers, targeting interpersonal skills, accounting for partner type and intimacy dynamics for regular sexual partners); and (3) take into account their exogenous environments (stigma of being MCMSW in West Africa).

## Conclusions

Little is known about male clients of male sex workers (MCMSW) in West Africa. Our results highlighted that a low proportion of MSM reported giving money, accommodation or gifts in exchange for sex (13.6%) and that this sub-population was characterized by older age, a lower educational level, unstable housing and exclusively insertive anal sex. A majority of them employed HIV risk-reduction strategies with male sexual partners, including engaging in protected anal sex, avoidance of sex when consuming psychoactive products, and practising exclusively insertive anal sex.

Despite these positive findings, our study also highlights the need for further research in West Africa targeting MCMSW and their practices, something that has been almost completely neglected to date. A better understanding of this sub-population could help reduce HIV transmission in West Africa.

## Supporting information

S1 Appendix Flow diagram on strategies used for selecting participants of our analysis. (DOCX)

S2 Appendix Variables of HIV risk-reduction strategies. (DOCX)

S3 Appendix Variables used to construct stigmatization scores. (DOCX)
