# Supplementary material for: Male clients of male sex workers in West Africa: A neglected high-risk population
Source: PLoS One. 2019 May 1;14(5):e0212245. doi: 10.1371/journal.pone.0212245 (PMC6493710; doi:10.1371/journal.pone.0212245)
Supplement: S1 Appendix — (DOCX) [file pone.0212245.s001.docx]

**S1 appendix**: Flow diagram on strategies used for selecting participants of our analysis
