# Supplementary material for: Male clients of male sex workers in West Africa: A neglected high-risk population
Source: PLoS One. 2019 May 1;14(5):e0212245. doi: 10.1371/journal.pone.0212245 (PMC6493710; doi:10.1371/journal.pone.0212245)
Supplement: S2 Appendix — (DOCX) [file pone.0212245.s002.docx]

**S2 appendix**: Variables of HIV risk-reduction strategies

| You sometimes avoid having sexual partners to reduce the risk of HIV infection |
| --- |
| You sometimes limit yourself to mutual masturbation, caresses or oral sex during sexual intercourse to reduce the risk of HIV infection |
| You sometimes avoid penetrating the anus of your partners to reduce the risk of HIV infection |
| You sometimes avoid one-night relationships to reduce the risk of HIV infection |
| You sometimes limit the number of partners with whom you have sex to reduce the risk of HIV infection |
| You sometimes ask your partners about their HIV status to reduce the risk of HIV infection |
| You sometimes avoid having sexual relations when you are drunk or you have consumed drugs to reduce the risk of HIV infection |
| You sometimes withdraw before ejaculation during condomless anal penetration to reduce the risk of HIV infection |
| You sometimes have condomless sexual relations with seropositive partners who you know are on ARV treatment, to reduce the risk of HIV infection |
| You sometimes have condomless sexual relations with seropositive partners who you know have an undetectable viral load, to reduce the risk of HIV infection |
| You sometimes use your HIV-positive partner’s, friend’s or purchased ARV treatment before you have sex to reduce the risk of HIV infection |
| You sometimes have post-exposure HIV prevention treatment (emergency treatment) after sexual intercourse to reduce the risk of HIV infection |
| You sometimes avoid penetrating the anus of seropositive partners or partners whose serostatus you do not know, to reduce the risk of HIV infection |
| You sometimes avoid being penetrated anally by seropositive partners or partners whose serostatus you do not know, to reduce the risk of HIV infection |
