# Supplementary material for: Male clients of male sex workers in West Africa: A neglected high-risk population
Source: PLoS One. 2019 May 1;14(5):e0212245. doi: 10.1371/journal.pone.0212245 (PMC6493710; doi:10.1371/journal.pone.0212245)
Supplement: S3 Appendix — (DOCX) [file pone.0212245.s003.docx]

**S3 appendix**: Variables used to construct stigmatization scores

| Experienced stigmatization score | During the past 6 months, have you been subjected to moral harassment (e.g., looks of disapproval) because of your sexual orientation? |
| --- | --- |
|  | During the past 6 months, have you been subjected to verbal abuse (insults, mockery) because of your sexual orientation? |
|  | During the past 6 months, have you been physically abused (beaten, stones thrown) because of your sexual orientation? |
|  | In the past 6 months, have you been coerced into forced sex (rape)? |
|  | In the past 6 months, have you been sexually blackmailed? |
| Perceived stigmatization score | Many people are reluctant to accept MSM |
|  | MSM would lose their female partners if they revealed their sexual orientation |
|  | Many employers underestimate a man because of his sexual orientation regardless of his qualification |
|  | Many people treat MSM differently than other people |
|  | Many people have negative attitudes towards MSM |
|  | Many people do not see MSM as normal people |
|  | MSM are not welcome at public meetings, for example, at parties, nightclubs or bars |
|  | Many families would be disappointed to have a homosexual son |
|  | Many people think that MSM are infected with HIV and will die of AIDS |
|  | Many people believe that MSM have several partners at the same time |
|  | Many health workers often exhibit an unpleasant or negative attitude when providing care to MSM |
| Internalized stigmatization score | Sometimes you regret having sex with men |
|  | Sometimes you think that if you were not attracted to men, you would probably be happier |
|  | To avoid revealing your sexual orientation, you have tried not to be attracted to men |
|  | Sometimes you would like to be sexually attracted only to women |
|  | In your opinion, you believe that homosexual practices is a personal defect |
|  | Sometimes you are ashamed of your sexual orientation |
|  | You are (were) afraid when your family and friends discover (ed) your sexual orientation |
|  | You try to appear heterosexual to avoid rejection by others |
